# Non-Invasive Biomarkers for Differentiating Alcohol Associated Hepatitis from Acute Decompensation in Patients with ALD

**DOI:** 10.3390/jcm13133747

**Published:** 2024-06-27

**Authors:** Mina Ignat, Horia Stefanescu

**Affiliations:** 1 Regional Institute of Gastroenterology and Hepatology “Prof. Dr. O. Fodor”, 400394 Cluj-Napoca, Romania; ignat.mina@gmail.com; 2 Faculty of Medicine, University of Medicine and Pharmacy “Iuliu Hatieganu”, 400347 Cluj-Napoca, Romania

**Keywords:** alcohol-associated hepatitis, decompensated cirrhosis, diagnosis, non-invasive biomarkers

## Abstract

Alcohol-associated hepatitis (AH) is the most severe form of alcohol-related liver disease. The natural course of alcohol-related liver disease is influenced by heavy alcohol consumption and abstinence periods. Differentiating between AH and decompensated cirrhosis (DC) could be extremely challenging in clinical practice due to clinical and bioclinical similarities. The severity of AH is made on bioclinical grounds, the severe form necessitating corticotherapy treatment. Liver biopsy is still the standard of care for establishing the diagnosis in atypical presentations. The pathogenesis of AH is an interplay between gene expression, cytokine dysregulation, the immune system and the gut microbiota. Non-invasive tests are increasingly and widely used for the purpose of early diagnosis and reliable prognostication. The non-invasive tests are emerging in concordance with disease pathogenesis. In this review, we describe the non-invasive tools that can distinguish AH from DC. We outline the available cut-offs and their performance in diagnosis and prognosis, as well as in assessing the treatment response to corticotherapy. Promising circulating biomarkers like keratin 18, microRNAs and sphingolipids will be in the review.

## 1. Introduction

Due to alcohol consumption in any pattern, more than 75 million adults are at risk of alcohol-related liver disease (ALD). ALD is the main cause of cirrhosis, being the third leading cause of mortality in people between 45 and 64 years old [1]. The occurrence of advanced liver disease is nearly 10–20% depending on the alcohol consumption pattern and the coexistence with metabolic factors and genetic predisposition [2,3]. Alcohol-related liver disease (ALD) is a broad spectrum of disorders, histologically classified as alcoholic fatty liver (AFL), alcoholic steatohepatitis (ASH), alcoholic cirrhosis (AC) and the most severe form, alcoholic-related hepatitis [4]. AH, in its severe form, is the fastest fibrosis route and has high short-term mortality (20–50% at three months) [5]. The lesions in ALD can occur separately, simultaneously or sequentially. The decompensation of cirrhosis is a complication of portal hypertension, systemic inflammation and impaired liver function [2]. The acute decompensation of cirrhosis encounters the development of ascites, hepatic encephalopathy, gastrointestinal bleeding and jaundice [6].

The Predict study identified two major precipitating events for DC: bacterial infection and alcohol-related hepatitis [7]. Differentiating between AH and other hepatic decompensations can be difficult. A clinical syndrome of jaundice and liver failure can be present in both DC and AH [8]. Furthermore, the AST/ALT ratio in AH is greater than 1.5 but can also be elevated in advanced cirrhosis, independently of the etiology [9].

Disease progression is interconnected with the natural history of alcoholism (profile of the consumption, abstinence and relapse events) and genetic factors, like variation in the PNPLA3 gene.

The diagnosis of ALD remains mainly clinical–pathological, with liver biopsies performed in some cases. However, differentiating AH from the acute decompensation of alcoholic cirrhosis can be extremely difficult because they have similar clinical features and biochemistry; therefore, liver biopsy is considered the gold standard of diagnosis of AH. Although being considered the gold standard for establishing a definite diagnosis, assessing the fibrosis stage and providing useful information for prognoses and treatment strategies, liver biopsy is associated with significant morbidity (especially as these patients have severe coagulopathy and/or ascites), high costs and expertise (as the trans-jugular route is often needed) and therefore is not widely used [10]. In contrast, non-invasive tools are easy to use, not difficult to repeat, more accessible and less expensive [11]. It is critical to differentiate between AH and DC because the therapeutic approach is different. Corticotherapy is extensively used to treat severe forms of AH, reducing the risk of death in the short term [12]. There are no medical resources for patients that are non-responders to therapy. In such cases, the only solution is early liver transplantation [13].

**Figure 1:**
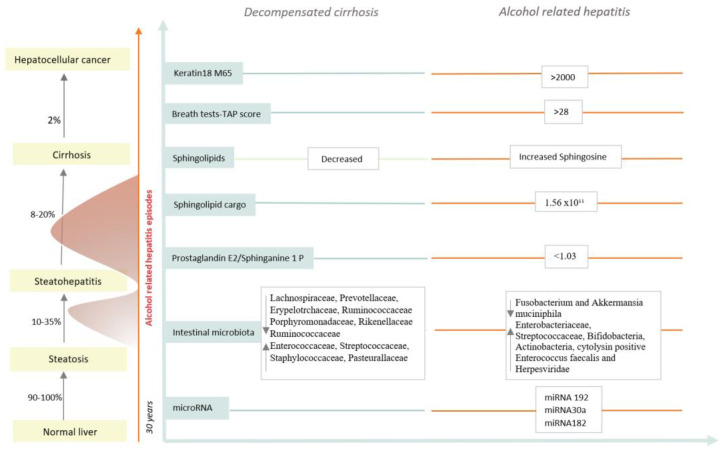
Natural evolution of alcohol-related liver disease. Heavy alcohol consumption leads to the development of different stages of the disease, from steatosis to hepatocellular carcinoma. The development of alcohol-related hepatitis episodes accelerates the fibrosis progression; 70% will develop liver cirrhosis. Circulating biomarkers for differentiating decompensated cirrhosis from AH are shown. Figure adapted from Nature Reviews [14].

Therefore, using proper non-invasive tools is necessary, especially in atypical cases, for the identification of the disease and the assessment of severity in order to make a treatment decision. From all the above, one can conclude that there is an unmet need for effective methods for differentiating AH from the acute decompensation of cirrhosis. Here, we review the non-invasive methods available to date and highlight future perspectives in the field (see Figure 1).

## 2. Natural History of ALD

### 2.1. ALD Early Stages

The development and progression of ALD are dependent on the level of alcohol consumption; however, only a small proportion of patients will develop the progression of the disease. Chronic alcohol consumption alters fat metabolism, induces lipolysis and increases fatty acids circulation, and, additionally, fat accumulates in hepatocytes [14]. Heavy alcohol abuse leads to the development of fatty liver and ASH. The histological pattern of alcohol-related injury consists of macrovesicular steatosis, lobular inflammation, hepatocellular ballooning and necrosis in the central portion of hepatic lobules [4]. ALD can be divided into an early stage (subclinical) and an advanced stage (clinical). Subclinical stages include fatty liver disease (steatosis), steatohepatitis (ASH) and compensated cirrhosis. Advanced stages incorporate AH, acute on chronic liver failure (ACLF) and decompensated cirrhosis [15].

The early screening of AFP is based on abdominal ultrasonography; additional imaging methods such as computed tomography and magnetic resonance imaging (superior, but too expensive for population screening) can also be used [16]. Ultrasound tools derived from the attenuation of shear waves, such as the controlled attenuation parameter (CAP), are preferred for steatosis screening [17]. On a histological level, fatty liver disease consists of macrovesicular accumulation localized in the centrilobular area of the hepatic lobules. The specific lesion area is explained by the fact that ethanol-metabolizing enzyme systems are richer in centrilobular areas compared to the periportal area [4]. The key element in the pathogenesis of ASH is inflammation. ASH will develop in 10–35% of individuals with ongoing excessive alcohol consumption and can only be accurately diagnosed with liver biopsy. From a histological point of view, ASH is marked by steatosis and hepatocellular injury: ballooning, Mallory Denk bodies, neutrophilic granulocytes and different levels of fibrosis with a pericellular pattern. Persistent hepatic injury and inflammation leads to the extension of cellular fibrosis, septa formation and the disruption of the lobular architecture. In total, 8–20% of ASH patients will further develop liver cirrhosis [4,15].

Alcohol-related hepatitis characterized by the rapid onset of jaundice is the most severe phenotype of ALD, causing a high risk of infections, ACLF development and multiorgan failure [18]. The diagnosis of alcoholic hepatitis is made on clinical grounds based on an alcoholism history and liver suggestive chemistry. The defined criteria for alcoholic hepatitis are the appearance of jaundice 8 weeks before the presentation, alcohol consumption of >40 g/day in women and >50–60 g/day in men with an abstinence period <60 days before the onset of jaundice, a serum bilirubin level greater than 3 mg/dL and an AST/ALT ratio >1.5, AST > 50 U/L. Liver biopsy is needed for diagnostic purposes in cases of atypical scenarios: uncertain alcohol use, uncommon laboratory tests (AST or AST > 400 U/L), hepatotoxic substance use in the last 3 months or a high suspicion of autoimmune hepatitis [19]. Severe AH is defined mainly by two prognostic scores: a modified discriminant function score greater or equal to 32 or a Model for End-Stage Liver Disease score greater than 20 [10,18,20]. Histological features can provide prognostic information with the aid of several scores. The AHHS score, taking into account the stage of fibrosis, bilirubinostasis, PMN infiltration and the presence of megamitochondria, is able to predict 90-day mortality in AH patients [21]. Additionally, veno-occlusive lesions, a consequence of intima proliferation and the obliteration of the vascular lumen of hepatic veins, are described in AH biopsies. Liver tissue infiltration by neutrophils gives a favorable outcome, probably in relation to a good regenerative response, meaning the patients are in a less advanced stage of cirrhosis. Furthermore, PMN infiltration activates cytokine secretion, which further stimulates liver regeneration [21,22].

A recent system for grading and staging fibrosis was developed in order to evaluate the prognostics of all spectra of the ALD disease. The macrovesicular steatosis, hepatocellular ballooning, lobular neutrophils and cholestasis were assessed by a numerical scoring system. The Salve scoring system describes seven fibrosis groups, four pre-cirrhotic groups and three stages of cirrhosis: with thin septa, broad septa and very broad septa, carrying different prognoses. For long-term survival, in the ALD spectrum, severe cirrhosis is an independent factor for poor outcomes [23].

### 2.2. Alcohol-Related Liver Cirrhosis

Histologically, cirrhosis is defined as architectural changes in the liver, regenerative nodules surrounded by fibrotic tissue [24]. The development of clinically significant portal hypertension leads to the occurrence of clinically decompensated events such as ascites, variceal bleeding, jaundice and hepatic encephalopathy (HE), transitioning from a compensated stage to a decompensated stage. Recently, based on the severity of the decompensation event, cirrhosis decompensation was stratified in acute decompensation, with the potential for ACLF development and non-acute decompensation. Acute decompensation is marked by the acute development of one major complication: ascites grade 2 or 3, variceal bleeding, first or recurrent HE and bacterial infection within a few weeks. In the non-acute stage, the occurrence of decompensation takes a slower rhythm; it is within months to years before the symptoms are severe and require hospitalization. The first decompensated event is usually non-acute [6]. A study describes three clinical courses of acute decompensation: pre-ACLF patients who develop ACLF with a high 3-month mortality, unstable acute decompensation needing frequent readmissions not related to ACLF and stable acute decompensation, which does not necessitate hospital readmission [7]. Pre-ACLF and unstable AC are susceptible to further decompensation; the pathogenetic mechanism seems to be the progression of systemic inflammation in pre-ACLF portal hypertension for unstable AD patients.

Non-invasive assessments of hepatic decompensation encompass: biochemical markers, elastography tools and imagistic instruments. Over the last years, the interest changed, originating at defining cirrhosis from a histological point of view for the identification of patients at risk for developing complications of portal hypertension. Clinically, significant portal hypertension (CSPH) is a stage when chronic liver damage and portal hypertension are developing, being at risk for decompensation. Liver stiffness measurements can diagnose clinically significant portal hypertension. With the development of non-invasive tests and the Baveno consensus in 2015, patients could safely avoid screening endoscopy if they fulfilled the following: liver stiffness measured by transient elastography < 20 kPa and a normal platelets count [11,25,26]. Nevertheless, dynamics in the liver stiffness measurement can provide prognostics; a 20% increase in liver stiffness raises the risk for liver decompensation by 50% [27].

The diagnosis of ascites is often clinically overt, and grade 1 ascites is often detected at regular ultrasonography reevaluations. A small amount of ascites is not considered decompensation but has a poorer prognostic [28]. Hepatic encephalopathy is made on clinical grounds. Despite many limitations regarding cut-off levels, there is no upper limit of normal; the ammonia level can be useful for ruling out HE, if normal. For covert hepatic encephalopathy, several tests are recommended, such as the gold standard (the psychometric score), the animal naming test and the Stroop test [29].

#### Predicting Decompensation

Fib-4 is extensively used in primary care for predicting significant fibrosis, combining four elements: AST, ALT, platelets and age [30]. Repeating FIB-4 measurements over time can identify patients at risk for severe liver disease. An increase in FIB-4 values during 5 years is associated with severe disease [3]. Also, sarcopenia (the loss of muscle mass) and frailty (loss of muscle function) can predict liver decompensation and ACLF development [31]. Liver stiffness measurements are able to predict liver decompensation. Ultrasound elastography can predict liver-related events in ALD better than the fibrosis stage on biopsy. A recent study showed that the hazard ratio for liver-related events is eight times greater if the liver stiffness is between 10 and 15 kPa. For a liver stiffness greater than 15 kPa, the hazard ratio is 28 [32]. The risk of decompensation follows the liver stiffness up to the value of 25 kPa; afterwards, no further increase in the risk is observed [33]. An enhanced liver fibrosis test (ELF test) can be a useful prognostic tool in secondary care, based on the correlation with advanced fibrosis, with an AUC of 0.87 in predicting liver outcomes [34].

## 3. Non-Invasive Assessment in Alcoholic-Related Hepatitis

### 3.1. Cross-Section Imaging in AH

Cross-sectional imaging is unnecessary for the diagnosis of AH; thus, it is usually performed for other reasons. AH may be associated with a specific imaging pattern, illustrated as a pseudotumoral appearance on computed tomography, and can mimic tumoral infiltrations. Heterogeneous steatosis (87% specificity) combined with transient perfusion disorder is a very specific feature of AH. Heterogeneous steatosis is defined by a geographical pattern, with a patchwork look of the liver. Transient hepatic perfusion disorders refer to regions of hepatic parenchyma only visible during the hepatic arterial phase. These lesions are described as hypo-attenuated areas in the pre-contrast phase and hyper-attenuated areas in the arterial phase, possibly in relation to high tissue regeneration. The association of heterogeneous steatosis and transient perfusion disorders has a great specificity, nearly 100%, for AH on CT and MRI. These characteristics were not found on ALD decompensated patients without AH, and the features tend to regress on the follow-up imaging. Although steatosis can be observed in patients with all stages of ALD and steatotic liver disease, heterogeneous steatosis is more frequent in patients with AH. Transient hepatic perfusion disorders must be interpreted in relation to the histological findings, canalicular and ductular cholestasis and the presence of ductular thrombi, which can lead to perfusion disorders via impaired liver portal perfusion. Other features, like homogeneous steatosis and an enlarged liver volume, are not that specific for AH [35,36]. However, heterogeneous arterial phase liver enhancement on gadolinium-enhanced MRI was also described in autoimmune hepatitis and drug-induced liver injury [37].

### 3.2. Circulating Biomarkers in Alcohol-Associated Hepatitis

#### 3.2.1. Serum Keratin 18

Serum keratin 18 fragments, a component of Mallory body, correlates with histological findings and 90-day mortality and can be useful in predicting the corticosteroid response. During hepatocellular injury, C18 is delivered from necrotic cells into the blood, split and referred to M65 and M30 [38,39]. Cytokeratin 18 is elevated in patients with sustained alcohol consumption and is higher in cirrhotic patients with active alcohol consumption in the last 3 months compared with no alcohol consumption. Keratin hepatic expression grows with the hepatic injury. Using circulating fragments of cytokeratin 18, M65, with upper and lower limits of 2000 U/L and 641 U/L, AH could be ruled in or ruled out, with a positive predicted value of 91% and a negative predicted value of 88% (see Table 1). However, for patients with a result between 641 and 2000 U/L, a liver biopsy still needs to be performed. The M65/ALT ratio can be useful in distinguishing AH. Another study of 824 patients from the STOPAH trial revealed the fact that serum K-18 M30 and M65 were associated with 90-day mortality. Prednisone administration showed survival benefits in the group of patients with an M-30 value greater than 5 U/L, with no survival benefits below this limit. This aspect may suggest that M30 can determine the inflammation that seems to respond to CS [38,40]. Although DC patients have significant elevations in both M30 and M65, AH patients have even higher levels. Woolbright et al. showed that the M30/M65 ratio is superior to other prognostic scores, like MELD and ABIC. In addition, Vatsalya et al. found that M65 and M30 were superior to MDF, MELD, ABIC and GAHS [41].

#### 3.2.2. Sphingolipids

Alcohol-induced lipid dysfunction reduces liver cell oxidation and lipid transport, increasing lipogenesis, eventually leading to steatosis [53]. Lipids play an important role in mediating the immune response. An altered lipid composition dysregulates immune processes, leading to uncontrolled inflammation. ALD is characterized by the depletion of sphingolipids, sphingomyelin and ceramides. Sphingolipids are components of the plasma membrane, playing a structural role but also having a role in mediating innate and adaptive immune responses [54]. Lower levels of sphingomyelin (SM) correlate with higher fibrosis stages and higher rates of liver decompensation events. The end-product of SM, sphingosine 1 phosphate (S1P), is also reduced. In DC, the expression of genes involved in SM synthesis (SPLTC1, SGMS1, SGMS2) or breakdown (SMPD1, SMPD3, SGPL1) are downregulated [55]. Sphingomyelin is reduced in decompensated cirrhosis without ACLF, while cholesterol esters (CES) and lipophosphatidylcholine levels are decreased in CD-ACLF patients, with AUC values of 0.93-0.98, in concordance with the number of failures. CES are used in the synthesis of steroid hormones and for that reason, reduced levels may lead to adrenal insufficiency in patients with ACLF and poor outcomes [56,57]. In severe alcoholic hepatitis, increased lipolysis induced by insulin resistance, stress hormones activity and the sympathetic system all lead to an increase in the level of membrane components, sphingosine in HA and a reduction in phospholipids metabolites, resulting in plasma membrane remodeling [42,43,58]. The ratio of the prostaglandin E2/Sphinganine 1 phosphate seems to discriminate AH from DC (AUC 0.96), and this result is not influenced by the presence of infection [44].

Extracellular vesicles (EV) are membrane nanometer-sizes particles released by cells. The EV count and sphingolipid cargo can serve as circulating biomarkers in AH. Using a cut-off of 1.56 × 10^11^ particles/mL can differentiate AH from decompensated cirrhosis with a sensitivity of 0.94% and a specificity of 77% based on an ROC curve of 0.88. An enriched sphingolipid cargo > 5.38 × 10^11^ Evs/mL can predict AH severity and mortality [42].

#### 3.2.3. The Gut Microbiota

The liver is influenced by the gut barrier function and the gut microbiome. The gut epithelial barrier can be damaged by a dysbiotic microbiome, leading to the transportation of bacterial components to the liver, via the portal vein, an entity named the leaky gut [59]. This condition is thought to increase systemic inflammation. Microorganisms and their components recognized as pathogen-associated molecular patterns (PAMPS) may set off an immune response to PAMPS and aggravate organ dysfunction. The gut microbiota-derived plasma signature changes during the progression of liver disease (Table 2). In normal conditions, the gut composition and activity are modulated by the immune system, bile acids secretion and gut immune cell antibacterial products [60]. Alcohol consumption has a direct effect on the gut microbiota, changing the diversity by reducing the abundance of *Bacteroidetes* and increasing *Proteobacteria*. Also, the alcohol metabolite acetaldehyde may disrupt tight junctions [61]. Furthermore, gut microbiota change in alcohol cirrhosis with an abundance of *Proteobacteria* (*Enterobacteriaceae*, *Enterococcaceae*) and *Staphylococcaceae* and decreased *Lachnospiraceae*, *Ruminococcaceae*, *Veillonellaceae*, *Clostridoides* Cluster XIV and *Porphyromonadaceae* [62].

In decompensated stages and ACLF, there is a reduced amount of *Lachnospiraceae*, *Ruminococcaceae*, *Erysipelotrichaceae*, *Prevotellaceae*, *Porphyromonadaceae*, *Rikenellaceae* and an enrichment in *Enterococcaceae*, *Peptostreptococcaceae*, *Streptococcaceae*, *Staphylococcaceae*, and *Pasteurallaceae* [63]. In AH, there are plenty of *Enterobacteriaceae*, *Streptococcaceae* and *Actinobacteria* [65] and a markedly reduced level of *Akkermansia muciniphila* (Gram-negative anaerobe protecting the intestinal barrier function) [64]. Enriched in cytolysin, positive *Enterococcus faecalis* was associated with mortality in AH patients. *Fusobacteria* levels are higher in those with alcohol consumption, but there is a low abundance in AH [67,68]. Increases in intestinal Mammalian viruses, such as *Parvoviridae* and *Herpesviridae*, were observed in AH. The increase in *Herpesviridae* was associated with 90-day mortality [48]. Reduced fungal diversity, with an abundance of *Candida* species, was observed in ALD patients. Anti-serum *Saccharomyces cerevisiae* antibodies were associated with poor 90-day survival and more intestinal translocation, being a mark of the immune response against fungi [58].

#### 3.2.4. MicroRNAs

MiRNAs are a class of small non-coding RNA that inhibit the expression of their target genes [69]. In circulation, a considerable proportion of miRNA are associated with EVs, being more stable in this form and more resistant to RNAse activity. The extracellular associated miRNA signature can be a useful marker in diagnosing AH. miRNA 192 and miRNA 30a were significantly increased in patients with AH compared with healthy controls [45] (Table 1). In liver tissue, miRNA 182 has been proven to be highly expressed in AH in tandem with disease severity, correlating with short-term mortality. In experimental studies, the blockade of mi182 reduced liver injury and bile acid accumulation. MiRNA 182 correlates with Meld score bilirubin levels and bilirubinostatis evaluated on liver histological assessment. However, miRNA 182 levels are not significantly different between AH and DC [70]. Intestinal permeability depends on the tight junctions’ function, the major part being represented by Zonula Occludens 1 protein. miR 212 is highly expressed in the intestinal epithelial cells of patients with ALD. miRNA 212 overexpression downregulates ZO-1 translation, leading to the disruption of the tight junction integrity and promoting the leaky gut [71,72]. Compared to heavy drinkers, reduced serum and hepatic levels of miR-30b-5p, miR-20a-5p, miR-146a-5p and miR-26b-5p were observed in AH individuals. Their target genes are responsible for DNA synthesis and the G1/S transition of the mitotic cycle. The higher expression of these RNAs predicts short-term morality, explained by the liver’s impaired ability to regenerate [73]. Ethanol metabolism requires oxygen utilization, generating hypoxia in the pericentral region. Endothelin-1 expression (ET-1) rises in response to alcohol, acting like a vasoconstrictor in liver sinusoidal endothelial cells (LSEC). Releasing ET-1 from LSEC enhances chemokine expression and promotes the infiltration with monocytes, PMN and T cells from circulation into the liver. miR199 is responsible for the modulation of ET-1 in rat and human LSEC by targeting ET-1 expression. In human cells, both miR-199 and miR-155 play a role in ET-1 modulation [74].

#### 3.2.5. Breath Tests

AH patients seem to have a specific pattern of breathprint using selected ion flow mass spectrometry. High levels of trimethylamine (TMA), acetone and pentane were observed in patients with AH. In patients with liver disease without AH, six exhaled breath compounds were found: 2-propanol, acetaldehyde, acetone, ethanol, pentane and TMA [75]. Intestinal bacteria are implicated in the formation of trimethylamine (TMA), afterwards being transformed into TMA N oxides (TMAO) by the hepatic enzymes [76]. In chronic liver diseases, the ability to transform TMA into TMAO is altered [77]. Also, the translocation of bacteria-derived lipopolysaccharides may explain the increased levels of TMA. The TAP score uses the combination of these three compounds (TMA, acetone and pentane), after adjusting for infections, for the purpose of AH diagnosis, with a sensibility of 97% and a specificity of 72% for a TAP score of 28 [51].

#### 3.2.6. Genetic Markers

The Gs meld score combines the gene signature and MELD score of patients with AH, and it has been associated with survival, being able to discriminate between patients with good survival and patients with poor survival at 90 and 180 days [78]. PNPLA3 gene polymorphism increases the risk of alcohol liver injury, alcohol cirrhosis and hepatocarcinoma [46]. PNPLA3 was associated with elevated liver enzymes and with liver fat content, increasing the risk for steatosis [79,80].

The other two genes implicated in lipid metabolic processes, TM6SF2 and MBOAT7, are associated with the risk of alcohol-related cirrhosis, suggesting the importance of the dysfunctional lipid turnover as the main mechanism. Homozygosity for rs 738409:G in PNPLA3 could be a risk factor for severe AH, with the potential of influencing medium-term mortality. In patients with ongoing alcohol consumption, no difference in mortality regarding the genotype was observed. In contrast, abstinence was associated with better survival in heterozygote carriers of ra738409:G or non-carriers but with poor outcomes in homozygous carriers [81]. HSD17B13 rs 72613567:TA is a protective factor for alcohol cirrhosis occurrence and HCC. HSD17B13 acts like a retinol dehydrogenase, leading to the depletion of hepatic retinoic acid in chronic alcohol consumers. Retinoic acid depletion induces the expression of activator protein 1 transcriptional complex, which is linked with hepatic cell hyperproliferation and hepatic carcinogenesis. In the rs 72613567:TA variant, the activity of retinol dehydrogenase is absent or reduced, explaining the protective role in HCC development [46,82].

#### 3.2.7. Other Biomarkers

Multiple components of the complement system, made up of a considerable number of distinct proteins involved in innate immunity, are impaired in patients with severe AH [83]. The complement system acts through three different but interacting pathways to recognize and destroy pathogens and self-modified antigens. Serum collectin 11, implicated in the activation of the lectin pathway, can differentiate between severe AH and alcoholic cirrhosis. Also, lower levels of the C1q binding protein, playing a role in the inhibition of classical pathway activation, are associated with severe alcohol-related hepatitis (sAH), insinuating an enhanced activation of the complement system in AH. Other components of the lectin pathway were associated with 90-day mortality: MASP1 with AUC 0.91 and F2 with AUC 0.77 [52].

Dysregulated cytokine metabolism plays a key role in the pathogenesis of AH. IL-6 and IL-8 can drive inflammation, and THF alpha increases gut permeability and enhances oxidative stress. IL-8 produced by hepatocytes has a role in neutrophile chemotaxis. The serum levels of THF-alpha, IL-6 and IL-8 were higher in AH. Patients with serum IL6 > 38.66 pg/mL have a shorter mean survival at 6 months. [84]. Additionally, chemokine CCL-20 produced by macrophages and hepatic stellate cells are upregulated in AH, modulating LPS-induced liver injury and being associated with disease severity scores and endotoxemia [85]. Extracellular matrix serum markers, such as laminin and collagen type IV, are components of the basement membrane in the space of Disse, being able to diagnose AH. Using a cut-off of 4.1 UI/mL, laminin can diagnose AH with a sensibility of 90% and a specificity of 77%. Also, collagen type IV levels greater than 150 ng/mL have a good accuracy in AH (SE = 89%, SP = 77%) [50].

## 4. Non-Invasive Assessment of Corticotherapy Response

In patients with severe alcoholic hepatitis, circulating bacterial DNA levels predicted infection development within 7 days after CS initiation. In patients who developed an infection in the first 7 days of CS, the medium-term mortality was higher [86].

Microvesicles (MVs) are membrane-bound extracellular vesicles (0.1–1 um) that can circulate into the blood. MVs associated with T cells, macrophages, hematopoietic stem cells and hepatocytes were more abundant in the non-responder patients at admission; therefore, the MVs signature in the peripheral blood can predict the CS response. Also, MVs from the CD34 count can predict 1-month mortality in non-responders [87]. An increased abundance of MVs from hepatocytes reflects persistent hepatocellular injury, while increased MVs from the hematopoietic cell reflect the destruction of bone marrow. The severity of liver cirrhosis can lead to hematological and immunological dysfunction through decreased hematopoietic stem cells (HSCs), especially in advanced stages. There are several possible explanations: a chronic pro-inflammatory environment in cirrhosis negatively affects HSCs and the increased demand among bone marrow HSCs for continuous hepatic regeneration leads to persistent activation, proliferation and eventually exhaustion. The early apoptosis of CD34 generates high levels of MVs CD34, while the liver histology of non-responders shows lower levels of CD34 [88]. The degree of serum lipopolysaccharide levels (LPS), a marker of bacterial translocation, plays a role in corticotherapy responses. Individuals with LPS levels ≤ 1.3 EU/mL have a greater chance to respond to CS compared with individuals with LPS levels > 1.30. LPS levels did not discriminate between infection associated with systemic inflammatory response syndrome (SIRS) and SIRS without infection, but together with SIRS, they are a predictor of multiple organ failure [89].

## 5. Conclusions and Future Directions

The key elements in the pathogenesis of AH are gene expression, cytokines dysregulation, immune responses and the gut liver axis. There is an unmet need for reliable biomarkers in order to provide a proper non-invasive diagnosis. Molecular subtypes need to be investigated in order to provide precision medicine tailored to the patient phenotype. The differentiation between alcoholic hepatitis (AH) and acute decompensation (DC) in patients with alcohol-related liver disease (ALD) presents a significant clinical challenge due to overlapping clinical features and biochemistry. While liver biopsy remains the gold standard for diagnosing AH, its limitations in terms of invasiveness, cost and expertise necessitate the exploration of non-invasive biomarkers. Recent advancements in non-invasive biomarkers offer promising avenues for improving the diagnostic accuracy and prognostic assessment of AH. Finding new diagnostic molecules suitable for the underlying pathophysiology of the disease brings a new therapeutic skyline. Furthermore, new treatment therapies are emerging, targeting the inflammatory pathways (inhibition of IL-1, IL-1B and IL-22) [90,91,92], the gut liver axis (microbiome transplantation, probiotics and rifaximin administration), apoptosis and necrosis (Emricasan, Selonsertib) [90]. Focusing on healthy donor fecal microbiota transplantation, studies has shown an improvement in liver function and survival [91]. Fecal microbiota were also used in decompensated cirrhosis for recurrent hepatic encephalopathy, improving cognition and dysbiosis [92,93].

It is now clear that identifying non-severe or moderate alcohol-related hepatitis at an early stage is very important in order to provide early intervention, both psychological and pharmacological. This group of patients with a Maddrey score < 32 and a Meld score < 20 is not currently treated, regardless of the risk of fibrosis progression and high mortality rates, up to 6% at 28 days and 13% at 1 year [94]. Differentiating non-severe AH from DC can be even more challenging, due to the lack of jaundice. In such cases, definite diagnosis may only be obtained by liver biopsy, returning to the main idea that non-invasive biomarkers are very much needed in the clinical practice and should address the whole spectrum of the disease.

## Figures and Tables

**Table 1:** Diagnostic biomarkers in alcohol-associated hepatitis.

Classification	Biomarkers	Diagnostic Ability	References
Cytokeratin 18 components	Cytokeratin 18 M65	M65 > 2000 IU/L sensibility = 67%, specificity = 92% M65 < 641 IU/L sensibility = 93%, specificity = 62%	Atkinson et al. [40]
Sphingolipids	Ev sphingolipid cargo Sphingolipids Prostaglandin E2/Sphinganine 1 P	>1.56 × 10^11^ particles/mL, discriminate AH from DC sensibility = 0.92%, specificity = 0.94% high levels of sphingosine PGE2/S1P < 1.03 discriminate AH from DC, AUC = 0.96	Sehrawat et al. [42] Rachkonda et al. [43] Horhat et al. [44]
microRNAs	miRNA 192	AUC = 0.96 for distinguishing AH from controls	Momen Heravi et al. [45]
	miRNA 30a	AUC = 0.85 for distinguishing AH from controls	
Genetics	PNPLA3	Homozygosity for Rs 738409:G—risk factor for AH occurrences	Salameh et al. [46]
Microbiota	Cytolysin positivity Enterococcus faecalis Mammalian viruses	Cytolysin-positive *Enterococcus faecalis*, *Herpesviridae* and Anti serum *Saccharomyces cerevisiae* antibodies are associated with mortality	Duan et al. [47] Jiang et al. [48] Lang et al. [49]
Extracellular matrix	Laminin	90% sensibility and 77% specificity for the diagnosis of AH, using a cut-off of 4.1 UI/mL	Forrest et al. [50]
	Collagen type IV	89% sensibility and 77% specificity for the diagnosis of AH, using a cut-off of >150 ng/mL	
Others	TAP score Serum collectin 11	sensibility = 90%, specificity = 80% discriminate AH from AC discriminate between sAH and AC; AUC = 0.77	Hanouneh et al. [51] Taiwo et al. [52]

Abbreviations: AH, alcohol-associated hepatitis; DC, decompensated cirrhosis; AC, alcohol-related cirrhosis; AUC, area under the curve; PNPLA3, patatin-like phospholipase domain-containing protein3; Ev, extracellular vesicle; TAP, TMA and pentane score.

**Table 2:** Gut microbiota changes.

Comparation	Alterd Phyla	Taxa Enriched	Taxa Depleted	References
Alcohol consumption	↑ *Proteobacteria* ↓ *Bacteroidetes*			Tilg et al. [60]
Compensated and decompensated cirrhosis		*Enterococcaceae*	*Lachnospiraceae*	Bajaj et al. [63]
		*Peptostreptococcaceae*	*Ruminococcaceae*	
		*Streptococcaceae*	*Erysipelotrchaceae*	
		*Staphylococcaceae*	*Prevotellaceae*	
			*Porphyromonadaceae*	
			*Rikenellaceae*	
Alcohol-associated hepatitis		*Enteriobacteriaceae* *Streptococcaceae* *Bifidobacteria* *Actinobacteria*	*Akkermansia muciniphilia*	Grander et al. [64] Llopis et al. [65] Ciocan et al. [66]
